# Olfactory Mucosal Mesenchymal Stem Cell‐Derived Exosomal LncA2M‐AS1 Ameliorates Parkinson's Disease by Regulating Microglial Glucose Metabolic Reprogramming and Neuroinflammation via the CFL1/ROCK1 Axis

**DOI:** 10.1002/cns.71019

**Published:** 2026-07-10

**Authors:** Jiangshan Zhang, Guoshuai Yang, Yanhui Zhou, Dan Hou, Chuang Wang, Yujie Hu, Ying Xia

**Affiliations:** ^1^ Department of Neurology Central South University Xiangya School of Medicine Affiliated Haikou Hospital Haikou Hainan Province P. R. China; ^2^ Department of Neurosurgery Central South University Xiangya School of Medicine Affiliated Haikou Hospital Haikou Hainan Province P. R. China

**Keywords:** exosomes, lncA2M‐AS1, microglial metabolism, neuroinflammation, olfactory mucosa mesenchymal stem cell, Parkinson's disease

## Abstract

**Background:**

Parkinson's disease (PD), a common neurodegenerative condition, afflicts patients through the progressive degeneration of dopaminergic neurons and sustained neuroinflammation. This study investigates the role of olfactory mucosa‐derived mesenchymal stem cell (OM‐MSC)‐derived exosomes, particularly the long non‐coding RNA A2M‐AS1 (lncA2M‐AS1), in modulating microglial metabolism reprogramming and neuroinflammation in PD.

**Methods:**

A mouse PD model was established using MPTP injections. Animals received treatments including OM‐MSC‐derived exosomes knockdown for lncA2M‐AS1 or AAV‐mediated lncA2M‐AS1 overexpression. Motor function was assessed using the open field test and the apomorphine‐induced rotation test. Glycolytic metabolism was evaluated by measuring ECAR and OCR using Seahorse XFp Analyzer, and the expression of glycolytic proteins (GLUT1, HK2, PKM2, LDHA) via Western blot. Molecular analyses included qPCR, Western blot, Co‐IP, and ubiquitination assays that were performed to investigate the lncA2M‐AS1/CFL1/ROCK1 regulatory axis. Histological examinations involved immunohistochemistry for TH and IBA1. The expressions of lncA2M‐AS1 and ROCK1 were determined in serum obtained from individuals with PD and matched controls.

**Results:**

LncA2M‐AS1 is downregulated in PD patient serum and MPTP mice. OM‐MSC exosomal lncA2M‐AS1 suppressed microglial glycolysis, reduced pro‐inflammatory cytokine release, enhanced neuronal viability, and improved motor function in PD mice. Mechanistically, lncA2M‐AS1 directly binds to CFL1 mRNA, promoting ubiquitin‐mediated degradation of ROCK1 and inhibiting the CFL1/ROCK1 pathway. Knockdown of CFL1 or overexpression of lncA2M‐AS1 attenuated microglial activation and neuroinflammation, whereas ROCK1 overexpression reversed these protective effects.

**Conclusion:**

OM‐MSC exosomal lncA2M‐AS1 ameliorates PD pathogenesis by targeting the CFL1/ROCK1 axis to reprogram microglial glucose metabolism and suppress neuroinflammation, offering a novel therapeutic strategy for PD.

## Graphical abstract

LncA2M‐AS1 targets CFL1 mRNA and destabilizes ROCK1 via ubiquitination. LncA2M‐AS1 in OM‐MSC exosomes targets CFL1 mRNA, modulating the CFL1–ROCK1 pathway to reprogram microglial glucose metabolism, suppress neuroinflammation, and ultimately protect dopaminergic neurons and reduce neuroinflammation in PD mice.

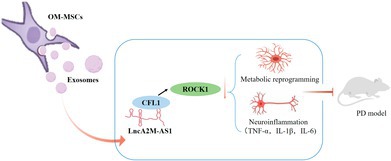

**Abbreviations**
2‐DG: 2‐Deoxy‐D‐glucose
AAV: Adeno‐associated virus
APO: Apomorphine
BSA: Bovine serum albumin
CCK‐8: Cell Counting Kit‐8
CFL1: Cofilin 1
Co‐IP: Co‐Immunoprecipitation
DAB: 3,3’‐Diaminobenzidine
DAPI: 4′,6‐diamidino‐2‐phenylindole
Drp1: dynamin‐related protein 1
ECAR: Extracellular Acidification Rate
ELISA: Enzyme‐linked immunosorbent assay
FCCP: Carbonyl cyanide 4‐(trifluoromethoxy)phenylhydrazone
GLUT1: Glucose transporter 1
HK2: Hexokinase 2
IBA1: Ionized calcium‐binding adapter molecule 1
IHC: Immunohistochemistry
IL‐1β: Interleukin‐1β
IL‐6: Interleukin‐6
LDHA: Lactate dehydrogenase A
lncA2M‐AS1: long non‐coding RNA A2M‐AS1
lncRNAs: long non‐coding RNAs
LPS: Lipopolysaccharide
miRNAs: microRNAs
MPTP: 1‐methyl‐4‐phenyl‐1,2,3,6‐tetrahydropyridine
MSCs: Mesenchymal stem cells
OCR: Oxygen Consumption Rate
oe‐NC: overexpression negative control
OM‐MSCs: Olfactory mucosa‐derived mesenchymal stem cells
PD: Parkinson's disease
PFA: Paraformaldehyde
PKM2: Pyruvate kinase M2
PLD1: phospholipase D1
qPCR: quantitative polymerase chain reaction
ROCK1: Rho‐associated coiled‐coil containing protein kinase 1
SD: Standard deviation
sh‐NC: negative control short hairpin RNA
shRNAs: short hairpin RNAs
TH: Tyrosine Hydroxylase
TNF‐α: Tumor necrosis factor‐α

## 1 Introduction

As the second leading cause of neurodegenerative disability worldwide, Parkinson's disease (PD) presents an escalating public health challenge, particularly in aging societies [1]. Its pathogenesis is driven by the progressive degeneration of nigral dopaminergic neurons and the presence of Lewy body pathology with aggregated α‐synuclein [2]. These lead to basal ganglia dysfunction and motor symptoms such as tremor, rigidity, and bradykinesia. Although current treatments like levodopa and deep brain stimulation can alleviate symptoms, they do not slow disease progression and often cause long‐term complications [3]. Thus, identifying novel disease‐modifying strategies that offer true neuroprotection or neural repair remains a paramount objective in PD research.

Neuroinflammation is a key driver of PD neurodegeneration [4]. As the innate immune sentinels of the central nervous system (CNS), microglia are pivotal for CNS homeostasis. In PD, their aberrant activation leads to the secretion of pro‐inflammatory mediators, which subsequently promote neuronal injury and compromise blood–brain barrier integrity [5]. Activated microglia shift their metabolic profile from oxidative phosphorylation to glycolysis. While this supports energy demands, sustained glycolysis leads to lactate and reactive oxygen species (ROS) accumulation, exacerbating neuroinflammation [6]. These insights suggest that reprogramming microglial glucose metabolism may represent a promising therapeutic strategy to alleviate neuroinflammation in PD.

Mesenchymal stem cells (MSCs), particularly olfactory mucosa‐derived MSCs (OM‐MSCs), offer therapeutic potential due to their multipotency, paracrine effects, and immunomodulatory capacity [7]. OM‐MSCs can be harvested minimally invasively, allow autologous transplantation, and exhibit neural differentiation potential [8]. Recent evidence suggests that many of the therapeutic benefits of OM‐MSCs may be largely mediated by their secreted exosomes—nanoscale vesicles that carry bioactive molecules such as proteins, microRNAs (miRNAs), and long non‐coding RNAs (lncRNAs). These exosomes can cross the blood–brain barrier and play essential roles in intercellular communication [9, 10]. Our previous study demonstrated that exosomes derived from OM‐MSCs are enriched with lncRNA A2M‐AS1 (lncA2M‐AS1), which mitigates oxidative stress and ameliorates behavioral deficits in PD models by interacting with IGF2BP1, thereby enabling its regulation of TP53INP1‐dependent mitophagy [11]. Nevertheless, whether and how lncA2M‐AS1 participates in the immunometabolic regulation of microglia remains incompletely understood.

Initial bioinformatic predictions pointed to a potential binding event involving lncA2M‐AS1 and the mRNA of Cofilin 1 (CFL1), a finding that offers an important research clue. Classified among the actin depolymerizing factors, CFL1 drives α‐synuclein aggregation and subsequent neuronal deficits in PD [12]. A subsequent exploration of interaction networks indicated a credible connection between CFL1 and Rho‐associated coiled‐coil containing protein kinase 1 (ROCK1). The latter serves as a major downstream executor of Rho GTPase signals and has emerging roles in controlling metabolic reprogramming events such as glycolysis [13]. In the neural context, ROCK1 promotes dopaminergic neuronal apoptosis in PD by activating dynamin‐related protein 1 (Drp1)‐mediated aberrant mitochondrial fission [14]. It is particularly noteworthy that CFL1 has been reported to sustain the expression of phospholipase D1 (PLD1) by inhibiting its ubiquitin‐mediated degradation [15], suggesting that CFL1 may similarly influence the stability of ROCK1 through an analogous mechanism.

Based on the aforementioned evidence, we propose that lncA2M‐AS1 in OM‐MSC exosomes targets CFL1 mRNA, modulating the CFL1–ROCK1 pathway to reprogram microglial glucose metabolism, suppress neuroinflammation, and ultimately protect dopaminergic neurons. This study aims to validate this mechanism, providing a foundation for lncA2M‐AS1‐based PD therapies.

## 2 Materials and Methods

### 2.1 Animals

Male C57BL/6 mice, 10–12 weeks of age, were housed under controlled environmental conditions with a temperature of 22°C ± 1°C and relative humidity of 55% ± 5%. A 12‐h light/dark cycle was maintained, with the light phase commencing at 07:00. Animals had ad libitum access to standard rodent diet and purified water. All experimental protocols were performed in compliance with the National Institutes of Health Guide for the Care and Use of Laboratory Animals and received approval from the Animal Ethics Committee of Haikou People's Hospital (ethics approval no. 2025–309).

### 2.2 Clinical Specimens

Serum samples were collected from 15 PD patients (8 males, 7 females; mean age 68.5 ± 6.2 years, range 58–79 years) and 15 age‐ and sex‐matched healthy controls (8 males, 7 females; mean age 67.8 ± 5.9 years, range 56–77 years). PD patients were diagnosed by board‐certified neurologists following the Movement Disorder Society Clinical Diagnostic Criteria for Parkinson's disease [16]. Inclusion criteria: Primary PD without other neurological disorders. Exclusion criteria: SecondaryParkinsonism, history of head trauma/stroke, severe systemic diseases, or use of anti‐inflammatory/immunosuppressive drugs within 3 months. Healthy controls had no neurological disorders or family history of PD. Peripheral blood was collected from fasting individuals and left to clot for 30 min at ambient temperature. Subsequent centrifugation was performed at 3,000 × g for 15 min at 4°C. The resulting supernatant was separated into aliquots and maintained at −80°C pending subsequent analysis.

### 2.3 Cell Culture

HT22 and BV2 cells (Procell Co.) were routinely cultured in complete DMEM (10% FBS, 1% penicillin–streptomycin) at 37°C under 5% CO_2_. An in vitro pathological environment was modeled by treating BV‐2 microglia with 200 ng/mL LPS [17].

Co‐culture was performed in a 0.4 μm Transwell system (Corning, USA). The lower chamber received pre‐treated BV2 cells, while the upper chamber received HT22 cells 12 h later. Following 48 h of co‐culture, HT22 cells were carefully collected for viability assessment using the CCK‐8 assay and for analysis of inflammatory cytokine levels via ELISA kits.

### 2.4 Cell Transfection

To knock down lncA2M‐AS1 or CFL1 expression, short hairpin RNAs (shRNAs) targeting lncA2M‐AS1 (sh‐A2M‐AS1) or CFL1 (sh‐CFL1) were synthesized by VectorBuilder (Guangzhou, China), along with a negative control shRNA (sh‐NC). For overexpression, the full‐length sequences of lncA2M‐AS1 or ROCK1 were cloned into the pcDNA3.1 vector (Youbio, China), with the empty vector serving as the overexpression negative control (oe‐NC). For transfection, cells were seeded into six‐well plates and transfected using Lipofectamine 3000 (Thermo Fisher Scientific, Massachusetts, USA) according to the manufacturer's instructions when reaching 60% confluency. After 48 h, transfection efficiency was validated via quantitative polymerase chain reaction (qPCR), and cells were harvested for subsequent experiments.

### 2.5 qPCR

Total RNA was purified from either brain tissue samples or cultured cells with a commercially available RNA isolation kit (Vazyme, Nanjing, China). Reverse transcription was performed with PrimeScript RT Master Mix (Biomed, Shanghai, China) to generate cDNA. QPCR amplification was conducted using SYBR Green Master Mix (Biomed) on an ABI7900‐HT‐Fast platform (Applied Biosystems, USA). Gene expression quantification was analyzed via the 2‐^ΔΔCt^ method, and all primer sequences utilized in this study are provided in Table 1.

**TABLE 1:** Primers used in this study.

Genes	5→3′
A2M‐AS1 Forward	GCACCACACAGAAGTGATAGC
A2M‐AS1 Reverse	TGGGACTTGACCTGAATTGGG
ROCK1 Forward	TGAAAGCCGCACTGATGGAT
ROCK1 Reverse	TGCCATCTATTCATTCCAGCCA
β‐Actin Forward	TGTCCACCTTCCAGCAGATGT
β‐Actin Reverse	AGCTCAGTAACAGTCCGCCTAG

### 2.6 Establishment of PD Model and Grouping

PD model was established in 10–12‐week‐old C57BL/6 mice by intraperitoneal injection of MPTP (MedChemExpress, Shanghai, China) at 20 mg/kg once daily over a 14‐day period to induce neurotoxicity [11]. The study consisted of two experimental parts. The first experimental phase comprised four groups (*n* = 6 per group): Control, MPTP, MPTP + OM‐MSC‐Exo sh‐NC, and MPTP + OM‐MSC‐Exo sh‐A2M‐AS1. Control mice received an equivalent volume of saline, while all other groups were injected with MPTP to induce PD. Seven days after the final MPTP administration, mice in the latter two groups received a stereotaxic intracerebroventricular injection (right lateral ventricle) of exosomes (1.4 × 10^11^ particles) [10] derived from OM‐MSCs transfected with sh‐NC or sh‐A2M‐AS1, as previously described [18]. Control animals were injected with the same volume of saline using the same procedure. A stereotactic injection was administered at coordinates −0.6 mm posterior, −1.5 mm lateral, and −1.7 mm ventral to the bregma [19].

In the second part, mice were divided into four groups (*n* = 6): Control, MPTP, MPTP + AAV‐oe‐NC, and MPTP + AAV‐oe‐A2M‐AS1. The PD modeling procedure was identical to that in the first part. Mice in the AAV‐treated groups were stereotaxically injected into the right lateral ventricle with AAV‐oe‐NC or AAV‐oe‐A2M‐AS1 (synthesized by Hanheng Biotechnology, Shanghai, China) at the same coordinates as in the first part. A total volume of 1 μL was injected per side at an infusion rate of 0.2 μL/min [20]. After injection, the needle was left in place for 5–10 min to prevent backflow and then slowly withdrawn.

### 2.7 Animal Behavior Tests

Behavioral tests were performed between weeks 4 and 6 post‐modeling, i.e., at least one week after stereotaxic surgery. During this one‐week recovery period, all mice were monitored daily for general status (locomotor activity, fur condition, body weight, and feeding), and no surgery‐related behavioral abnormalities (e.g., pain, hypoactivity, or rotation) were observed. At the conclusion of week 6, animals were humanely euthanized for tissue harvesting. In the open field test, each mouse was released into the center of a square arena (50 × 50 × 40 cm) with opaque black walls. The floor of the arena was virtually divided into equal‐area squares. After a 30‐s acclimatization period with the chamber covered, the cover was removed, and rodent behavior was monitored for 3 min using an automated video tracking system. Recorded data were processed with Image OF software (O'Hara & Co. Ltd., Japan). For apomorphine (APO)‐induced rotation assessment, animals from different groups received subcutaneous administration of 0.5 mg/kg APO (Sigma‐Aldrich, United States). The total number of rotations made by each animal was recorded over a 30‐min period and compared across groups to evaluate motor impairment and treatment effects.

### 2.8 Immunohistochemistry (IHC)

After 24‐h fixation in 4% paraformaldehyde (PFA, Sangon, Shanghai, China) at 4°C, substantia nigra tissues were dehydrated, cleared in xylene, and embedded in paraffin. Sections (4 μm) were cut using a microtome (Leica RM2235), dewaxed, and rehydrated. Antigen retrieval was performed in citrate buffer (pH 6.0) at 95°C for 20 min. Endogenous peroxidase was blocked with 3% H₂O₂, and nonspecific sites were blocked with 5% bovine serum albumin (BSA, Sangon). Sections were incubated overnight at 4°C with anti‐Tyrosine Hydroxylase (TH, ab315252, Abcam, Cambridge, UK) or anti‐IBA1 antibodies (ab178846, Abcam), followed by HRP‐conjugated secondary antibody (Abcam, ab6721) for 1 h at 37°C. Staining was visualized using DAB (Sangon), and sections were counterstained with hematoxylin (Sangon). Five fields per section were imaged under a light microscope (Nikon Eclipse 80i) for semi‐quantitative analysis of TH‐positive dopaminergic neurons and IBA1‐positive microglia.

### 2.9 Enzyme‐Linked Immunosorbent Assay (ELISA)

Tissue homogenates were centrifuged to collect supernatants, and the concentrations of interleukin‐1β (IL‐1β), tumor necrosis factor‐α (TNF‐α), and interleukin‐6 (IL‐6) were quantified using commercial ELISA kits (Elabscience, Wuhan, China) in accordance with the manufacturer's instructions. Absorbance was measured with a microplate reader (DeTiebio, China).

### 2.10 Nissl Staining

Nissl staining was performed on 4 μm‐thick spinal cord tissue sections using a commercial staining kit (Beyotime). Following standard steps of fixation, rinsing, and dehydration, the sections were coverslipped. Nissl‐positive bodies were quantified under a light microscope.

### 2.11 Exosomes Uptake Assay

OM‐MSC‐derived exosomes were fluorescently labeled with PKH67 (Sigma Aldrich, USA) following the manufacturer's protocol. The labeled suspension was centrifuged at 300 × g for 15 min, after which the supernatant was discarded. The pellet was washed twice with PBS and subsequently cultured for 48 h. Exosomes were then isolated from the conditioned media of these OM‐MSCs and co‐cultured with BV2 cells at 37°C for 3 h. Following incubation, the cells were rinsed with PBS and fixed in 4% paraformaldehyde for 15 min. After additional PBS washes, cell nuclei were stained with DAPI (Invitrogen, USA). Fluorescence signals were visualized using a Leica DMI6000B microscope (Germany).

### 2.12 Cell Counting Kit 8 (CCK‐8)

Cells were seeded in 96‐well plates at a density of 3 × 10^3^ per well and cultured until 80% confluency was reached. The cells were then incubated with CCK‐8 reagent (Shyuanye, China) for 2 h. Absorbance at 490 nm was measured using a microplate reader.

### 2.13 Lactate Level Measurement

Lactate levels in BV2 cell culture supernatants were measured using a Lactate Assay Kit (BioVision, Milpitas, CA, USA) according to the manufacturer's instructions. Briefly, 50 μL of supernatant was mixed with the reaction buffer and enzyme mix, incubated at 37°C for 30 min, and the absorbance was read at 450 nm using a microplate reader. Lactate concentration was calculated from a standard curve and normalized to total protein concentration.

### 2.14 Real‐Time Cell Metabolism Assay

Cells were seeded at a density of 5 × 10^4^ per well in an XF96 cell culture plate and incubated overnight. The next day, the culture medium was replaced with Seahorse XF Base Medium (Agilent, USA) supplemented with 2 mM glutamine, 1 mM pyruvate, and 10 mM glucose (for OCR measurement) or without glucose (for ECAR measurement), and the plate was incubated in a non‐CO₂ incubator at 37°C for 1 h. Extracellular acidification rate (ECAR) and oxygen consumption rate (OCR) were measured using a Seahorse XFp Analyzer (Agilent). To assess glycolytic function, a Glycolysis Stress Test was performed with sequential injections of glucose (10 mM), oligomycin (1 μM), and 2‐deoxy‐D‐glucose (2‐DG, 50 mM) at the indicated time points. Glucose injection stimulates maximal glycolytic flux; oligomycin inhibits ATP synthase, shifting energy production to glycolysis and revealing glycolytic capacity; 2‐DG, a glucose analogue, inhibits hexokinase and terminates glycolysis, allowing calculation of non‐glycolytic acidification. For mitochondrial respiration, a Mitochondrial Stress Test was performed with sequential injections of oligomycin (1 μM), FCCP (1 μM), and a mixture of rotenone (0.5 μM) and antimycin A (0.5 μM). Oligomycin inhibits ATP‐linked respiration; FCCP uncouples mitochondrial membrane potential to reveal maximal respiration; rotenone/antimycin A inhibit complexes I and III, shutting down mitochondrial respiration and allowing calculation of non‐mitochondrial oxygen consumption. ECAR and OCR values were normalized to protein content per well.

### 2.15 Bioinformatics Analysis

Human LncA2M‐AS1 (ENSG00000245105) was queried in the ENCORI/starBase database (https://rnasysu.com/encori/), and CFL1 was identified as a potential interacting mRNA target. To identify potential interacting partners of CFL1, we employed the STRING database (https://string‐db.org/). The analysis was performed with the following parameters: Query protein = CFL1, organism = *Homo sapiens* , and a confidence threshold of combined score > 0.7 to ensure high reliability.

### 2.16 Dual‐Luciferase Reporter Assays

To investigate the potential direct targeting and binding between lncA2M‐AS1 and CFL1 mRNA, a dual‐luciferase reporter assay was performed. Cells were co‐transfected with either wild‐type (pGL3‐CFL1‐WT) or mutant (pGL3‐CFL1‐MUT) reporter plasmids, together with oe‐A2M‐AS1 or oe‐NC, using Lipo3000 transfection reagent. The pRL‐TK vector expressing Renilla luciferase was included in all transfections to normalize transfection efficiency. After 48 h, luciferase activity was assessed using a commercial dual‐luciferase detection system (Promega, USA). The relative luciferase activity was defined as the ratio of firefly to Renilla luciferase signals.

### 2.17 Co‐Immunoprecipitation (Co‐IP)

Cell lysates from BV‐2 cells were incubated with anti‐ROCK1 antibody (ab134181, Abcam) or IgG control overnight at 4°C, followed by addition of Protein A/G beads (Thermo Fisher) for 4 h. Beads were washed three times with cold RIPA buffer, and bound proteins were eluted with 2 × SDS loading buffer. Immunoprecipitated CFL1 was detected by western blot using anti‐CFL1 antibody (AF6232, Affinity, USA).

### 2.18 Ubiquitination

To evaluate ROCK1 ubiquitination, BV‐2 cells were transfected to express HA‐tagged ubiquitin. Following the designated treatments, the cells were exposed to 20 μM MG‐132 (MedChemExpress), a proteasome inhibitor, for 4 h to prevent the degradation of ubiquitinated proteins. Whole‐cell lysates were subsequently prepared and subjected to immunoprecipitation using an anti‐ROCK1 antibody (ab155282, Abcam), with normal IgG serving as the negative control. The immunoprecipitated complexes were then analyzed by western blot with an anti‐ubiquitin antibody (ab134953, Abcam) to detect ubiquitin‐conjugated ROCK1. Input samples were probed with anti‐ROCK1 to verify consistent protein loading.

### 2.19 RNA Stability Assay

RNA stability was assessed according to a previously described protocol [21]. Briefly, cells were cultured overnight in six‐well plates and then treated with 5 μg/mL actinomycin D (Abmole, China) to block transcription for the indicated time periods. mRNA levels were subsequently quantified via qPCR.

### 2.20 Western Blot

Protein extracts were prepared from brain tissues using RIPA lysis buffer (TargetMol, China). Protein concentration was determined with a BCA assay before equal amounts of lysates were separated on 10% SDS–PAGE gels (Solarbio, China) and transferred onto PVDF membranes (Yeasen, China). The membranes were blocked with 5% non‐fat milk (Yeasen) for 1 h at room temperature and then incubated with primary antibodies (Table 2) overnight at 4°C. After washing with TBST, the membranes were incubated with HRP‐conjugated secondary antibodies for 1 h at room temperature. Protein bands were detected with an ECL substrate (Life‐ilab, China) and imaged using an iBright FL1500 system (Invitrogen). β‐actin was used as a loading control.

**TABLE 2:** Antibodies used in this study.

Name	Catalog	Dilution	Manufacturer
GLUT1	ab150299	1/200	Abcam
HK2	ab209847	1/1000	Abcam
PKM2	AF5234	1/1000	Affinity
LDHA	DF6280	1/1000	Affinity
CFL1	AF6232	1/1000	Affinity
β‐Actin	ab8226	1/10000	Abcam
Goat anti‐rabbit	ab205718	1/10000	Abcam
Goat anti‐mouse	ab205719	1/10000	Abcam

### 2.21 Statistical Analysis

Statistical analysis was undertaken using GraphPad Prism software (Version 8.0, USA). The measurement data were shown as mean ± standard deviation (SD). The Student's *t*‐test was used for comparisons between two groups. Multiple groups were done through a one‐way analysis of variance followed by Tukey's *post hoc* test. In all statisticalanalyses, a level of significance of *p* < 0.05 was assumed.

## 3 Results

### 3.1 LncA2M‐AS1 Carried by OM‐MSC‐Derived Exosomes Alleviates Motor Deficits and Modulates Glycolysis and Neuroinflammation in a PD Mouse Model

**FIGURE 1:**
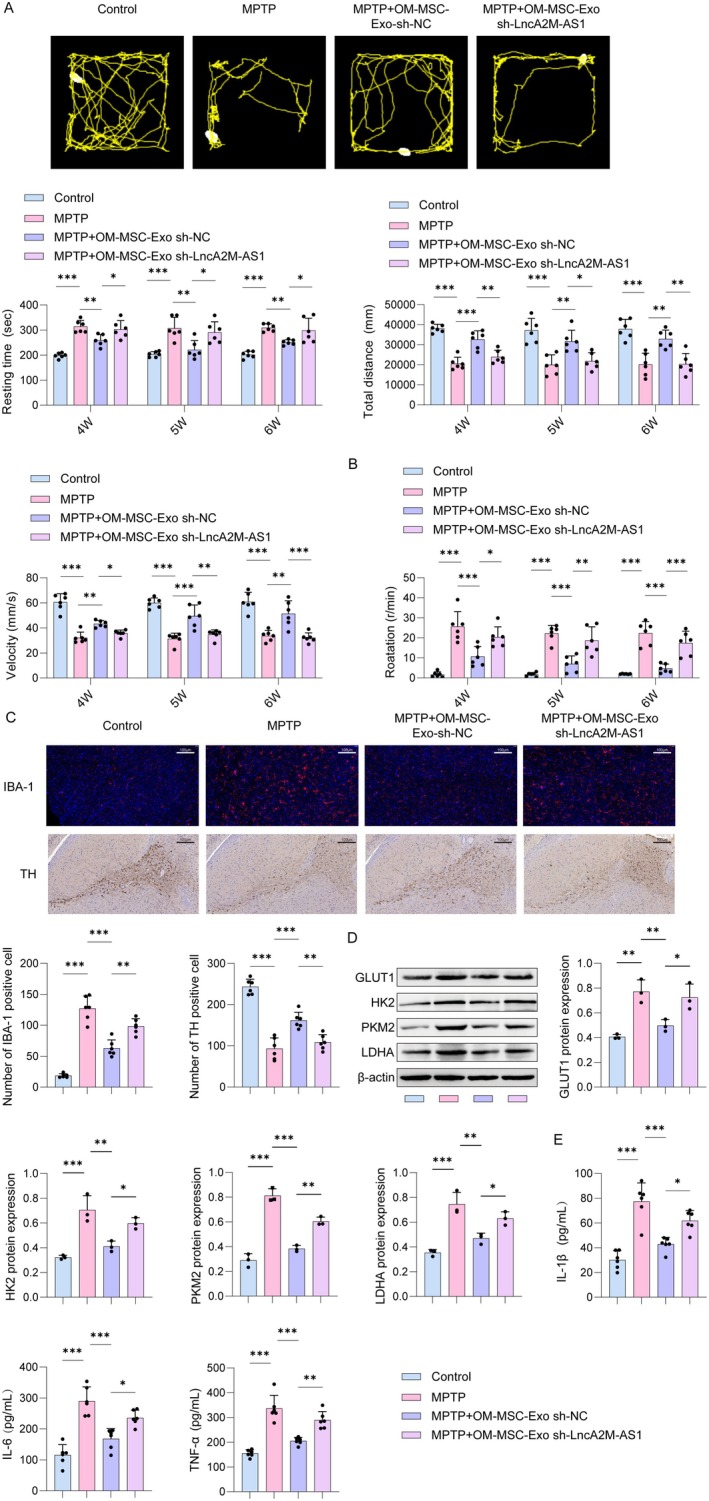
Effects of OM‐MSC‐Exo sh‐LncA2M‐AS1 in PD mice. (A) Open field test: Total distance moved, movement velocity, and resting time within 10 min at weeks 4, 5, and 6. (B) Apomorphine‐induced rotation bias at week 4. (C) Immunohistochemical staining of TH and IBA1 in the substantia nigra. (D) Western blot analysis of GLUT1, HK2, PKM2, and LDHA protein expression in brain tissue. (E) ELISA quantification of TNF‐α, IL‐1β, and IL‐6 levels. Data are presented as mean ± SD. *p < 0.05, **p < 0.01, ***p < 0.001.

Having previously established and characterized OM‐MSC‐Exo in our published work [11], we investigated its functional significance in a PD mouse model. Behavioral analyses indicated that administration of control exosomes (OM‐MSC‐Exo sh‐NC) to MPTP‐intoxicated mice resulted in improved motor parameters, including increased velocity, longer movement trajectories, and decreased resting time in the open field test (Figure 1A), along with a significant reduction in apomorphine‐induced rotations (Figure 1B). These behavioral improvements were partially reversed upon treatment with exosomes derived from lncA2M‐AS1‐knockdown OM‐MSCs (Figure 1A,B). Immunohistochemical analysis revealed that OM‐MSC‐Exo sh‐NC significantly increased TH expression and decreased IBA1 levels in the substantia nigra of MPTP mice, whereas exosomes from lncA2M‐AS1‐knockdown cells counteracted these effects (Figure 1C). Western blot analysis further demonstrated that OM‐MSC‐Exo sh‐NC downregulated key glycolytic proteins (GLUT1, HK2, PKM2, and LDHA) in brain tissues, an effect that was reversed by lncA2M‐AS1 knockdown (Figure 1D). Additionally, OM‐MSC‐Exo sh‐NC diminished pro‐inflammatory cytokines (TNF‐α, IL‐1β, and IL‐6) concentrations in MPTP mice, which was again attenuated by lncA2M‐AS1 knockdown (Figure 1E). These findings suggest that lncA2M‐AS1 encapsulated in OM‐MSC‐Exo may ameliorate PD progression by modulating glycolytic reprogramming and neuroinflammation.

### 3.2 LncA2M‐AS1 From OM‐MSC Exosomes Regulates Microglial Metabolic Activity and Inflammatory Response in Vitro

**FIGURE 2:**
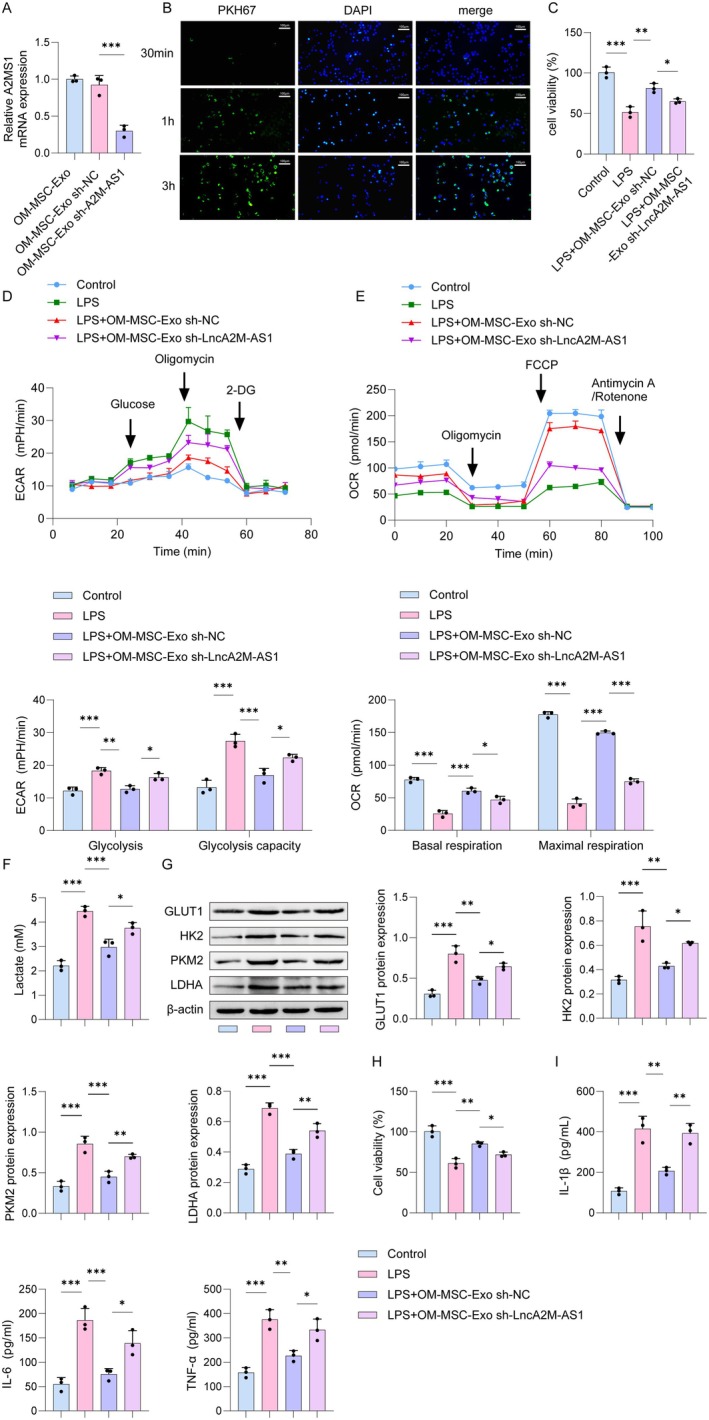
Uptake and functional influence of OM‐MSC‐Exo sh‐LncA2M‐AS1 on BV2 microglial cells. (A) qPCR detection of lncA2M‐AS1 expression in isolated OM‐MSC exosomes. (B) Uptake of PKH67‐labeled exosomes by BV2 cells observed via immunofluorescence. (C) BV2 cell viability assessed by CCK‐8 assay. (D) Extracellular acidification rate (ECAR) in BV2 cells. (E) Oxygen consumption rate (OCR) in BV2 cells. (F) Lactate levels were measured using the kit. (G) Western blot analysis of GLUT1, HK2, PKM2, and LDHA in BV2 cells. (H) Viability of HT22 cells co‐cultured with BV2 cells measured by CCK‐8. (I) Levels of TNF‐α, IL‐1β, and IL‐6 in HT22 cells co‐cultured with BV2 cells via ELISA. Data are presented as mean ± SD. *p < 0.05, **p < 0.01, ***p < 0.001.

To further elucidate the role of OM‐MSC exosomes, in vitro experiments were performed. qPCR confirmed successful knockdown of lncA2M‐AS1 in exosomes derived from OM‐MSCs (OM‐MSC‐Exo sh‐lncA2M‐AS1) compared to control sh‐NC exosomes (Figure 2A). Uptake of fluorescently labeled exosomes by BV2 microglial cells was observed after 3 h of co‐culture (Figure 2B). Under LPS‐induced inflammatory conditions, BV2 cells exhibited reduced viability (Figure 2C). Treatment with OM‐MSC‐Exo sh‐NC partially reversed these effects. These beneficial effects were abolished by lncA2M‐AS1 knockdown (Figure 2C). Under LPS‐induced inflammatory conditions, BV2 cells exhibited a marked increase in glycolytic flux, as evidenced by elevated basal ECAR following glucose injection, increased glycolytic capacity after oligomycin treatment, and reduced glycolysis inhibition by 2‐DG (Figure 2D). LPS stimulation also significantly decreased OCR parameters, including basal respiration, ATP‐linked respiration, and maximal respiration, indicating impaired mitochondrial oxidative phosphorylation (Figure 2E). Treatment with OM‐MSC‐Exo sh‐NC partially reversed these metabolic alterations: It reduced the LPS‐induced ECAR increase (lower basal glycolysis and glycolytic capacity), restored OCR parameters (improved basal and maximal respiration), and decreased lactate levels (Figure 2F). Furthermore, OM‐MSC‐Exo sh‐NC downregulated the expression of glycolytic proteins GLUT1, HK2, PKM2, and LDHA (Figure 2G). All these beneficial effects were abolished by lncA2M‐AS1 knockdown (OM‐MSC‐Exo sh‐LncA2M‐AS1), resulting in metabolic profiles similar to the LPS‐only group (Figure 2D–G). We further evaluated the viability and inflammatory cytokine (TNF‐α, IL‐1β, IL‐6) levels in HT22 neurons that had been co‐cultured with differently treated BV2 microglial cells. HT22 cells co‐cultured with LPS‐stimulated BV2 cells showed significantly reduced viability (Figure 2H) and increased secretion of inflammatory factors (Figure 2I). When BV2 cells were pre‐treated with OM‐MSC‐Exo before LPS stimulation, the viability of co‐cultured HT22 cells was restored (Figure 2H), and their inflammatory cytokine levels were significantly decreased (Figure 2I). However, these protective effects were abolished when BV2 cells were treated with OM‐MSC‐Exo sh‐LncA2M‐AS1 (Figure 2H,I). These results suggest that lncA2M‐AS1 delivered by OM‐MSC exosomes attenuates microglia‐mediated neurotoxicity and inflammatory responses in neurons, possibly through regulation of microglial glycolytic reprogramming and neuroinflammation.

### 3.3 LncA2M‐AS1 Inhibits ROCK1 Expression and Modulates Microglia‐Mediated Glycolysis and Inflammation

**FIGURE 3:**
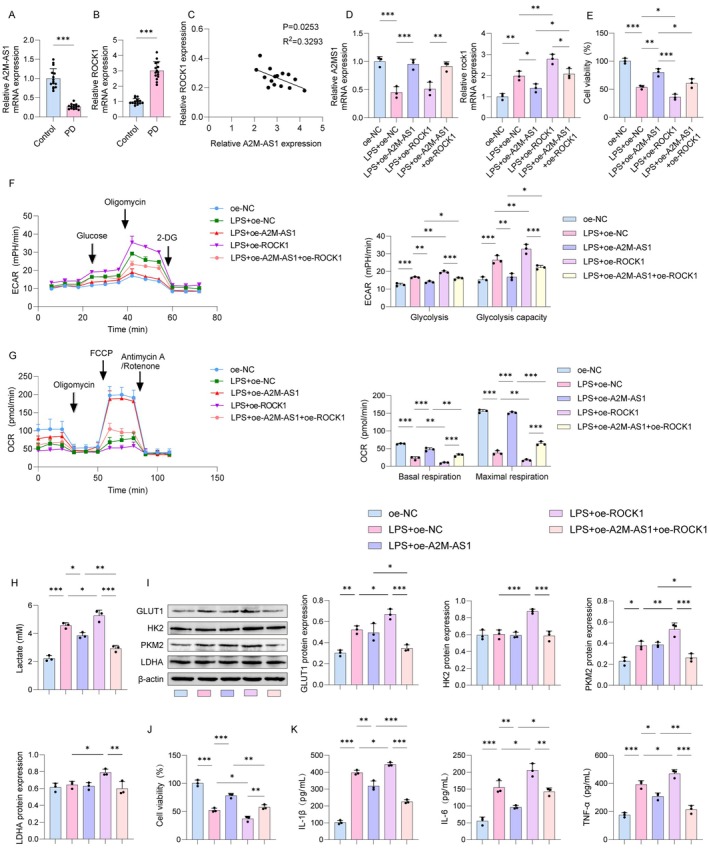
Expression and functional correlation of lncA2M‐AS1 and ROCK1 in PD. (A) LncA2M‐AS1 expression in serum from normal and PD patients by qPCR. (B) ROCK1 expression in serum from normal and PD patients by qPCR. (C) Correlation analysis between lncA2M‐AS1 and ROCK1 expression in PD. Fitted by simple linear regression. (D) qPCR detection of lncA2M‐AS1 and ROCK1 in BV2 cells across groups. (E) Viability of BV2 cells by CCK‐8 assay. (F) ECAR profiles of BV2 cells. (G) OCR profiles of BV2 cells. (H) Lactate levels were measured using a kit. (I) Western blot of GLUT1, HK2, PKM2, and LDHA in BV2 cells. (J) Viability of HT22 cells co‐cultured with BV2 cells. (K) TNF‐α, IL‐1β, and IL‐6 levels in HT22 cells co‐cultured with BV2 cells via ELISA. Data are presented as mean ± SD. *p < 0.05, **p < 0.01, ***p < 0.001.

Evaluation of clinical specimens demonstrated that lncA2M‐AS1 was downregulated and ROCK1 was upregulated in PD, with a negative correlation between their expression levels (Figure 3A–C). In LPS‐stimulated BV‐2 cells, lncA2M‐AS1 was downregulated, and ROCK1 was upregulated. Overexpression of lncA2M‐AS1 elevated its expression while reducing ROCK1 levels. Conversely, ROCK1 overexpression did not affect lncA2M‐AS1 but increased ROCK1 expression. Co‐transfection with oe‐ROCK1 and oe‐A2M‐AS1 reversed the individual effects of oe‐ROCK1 (Figure 3D). Additionally, compared to LPS‐induced BV‐2 cells, oe‐A2M‐AS1 not only promoted cell viability (Figure 3E), decreased ECAR and lactate levels (Figure 3F,H), and increased OCR (Figure 3G), but also suppressed the expression of glycolysis‐related proteins (Figure 3I). In contrast, oe‐ROCK1 exerted effects opposite to those of oe‐A2M‐AS1, and co‐transfection with oe‐ROCK1 and oe‐A2M‐AS1 counteracted their respective effects on cell viability, glycolysis, lactate levels, ECAR, and OCR in LPS‐induced BV‐2 cells (Figure 3E–I). Moreover, compared with HT22 cells co‐cultured with LPS + oe‐NC‐treated BV‐2 cells, those co‐cultured with LPS + oe‐A2M‐AS1 BV‐2 cells exhibited increased viability and decreased inflammatory cytokine levels, whereas HT22 cells co‐cultured with LPS + oe‐ROCK1 BV‐2 cells showed further reduced viability and increased inflammatory cytokine levels. Co‐transfection with oe‐A2M‐AS1 and oe‐ROCK1 reversed the effects on viability and inflammatory cytokine levels in HT22 cells induced by either oe‐A2M‐AS1 or oe‐ROCK1 alone (Figure 3J,K). These results indicate that lncA2M‐AS1 regulates microglial glucose metabolic reprogramming and neuroinflammation through ROCK1.

### 3.4 LncA2M‐AS1 Targets CFL1 mRNA and Promotes ROCK1 Ubiquitination and Degradation

**FIGURE 4:**
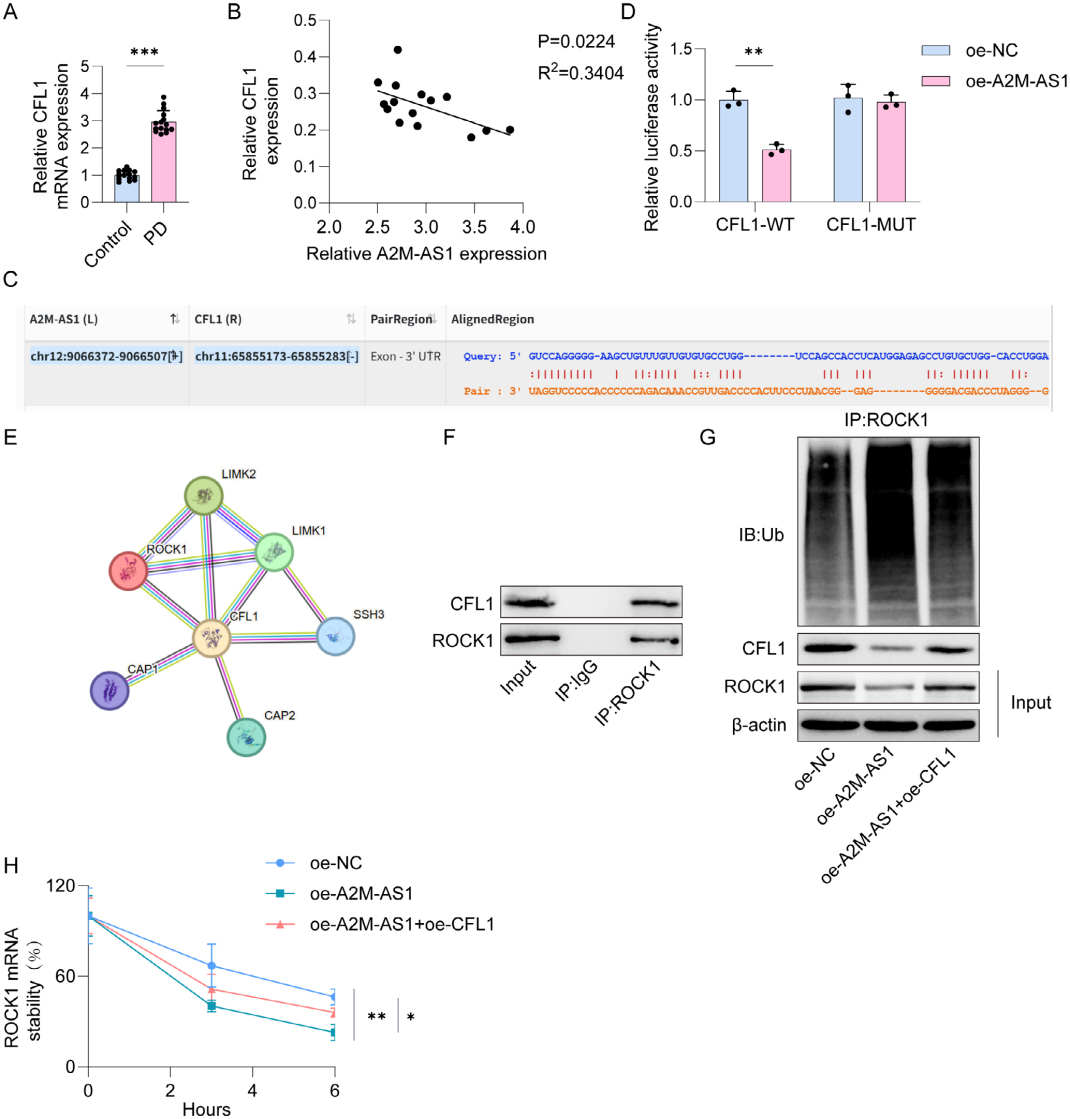
Molecular interactions among LncA2M‐AS1, CFL1, and ROCK1. (A) CFL1 expression in serum from normal and PD patients by qPCR. (B) Correlation between lncA2M‐AS1 and CFL1 expression in PD. (C) Predicted binding between lncA2M‐AS1 and CFL1 mRNA. (D) Dual‐luciferase reporter assay confirming lncA2M‐AS1 binding to CFL1. (E) Bioinformatic prediction (string‐db.org) of ROCK1 as a CFL1‐interacting protein. (F) Co‐immunoprecipitation of CFL1 and ROCK1. (G) Ubiquitination level of ROCK1. (H) ROCK1 mRNA stability assay using actinomycin D. Data are presented as mean ± SD. *p < 0.05, **p < 0.01, ***p < 0.001.

CFL1 was downregulated in PD patient serum and negatively correlated with lncA2M‐AS1 expression (Figure 4A,B). Bioinformatic analysis and dual‐luciferase reporter assays confirmed that lncA2M‐AS1 directly binds to CFL1 mRNA (Figure 4C,D). Using the STRING database, ROCK1 was predicted to interact with CFL1, which was validated by Co‐IP (Figure 4E,F). Overexpression of lncA2M‐AS1 in BV2 cells reduced both CFL1 and ROCK1 protein levels, increased ROCK1 ubiquitination (Figure 4G), and decreased ROCK1 mRNA stability (Figure 4H). These effects were partially reversed by simultaneous overexpression of CFL1. These findings indicate that lncA2M‐AS1 targets CFL1 and promotes post‐translational degradation of ROCK1.

### 3.5 Knockdown of CFL1 Suppresses Microglial Activation and Neuroinflammation via ROCK1

**FIGURE 5:**
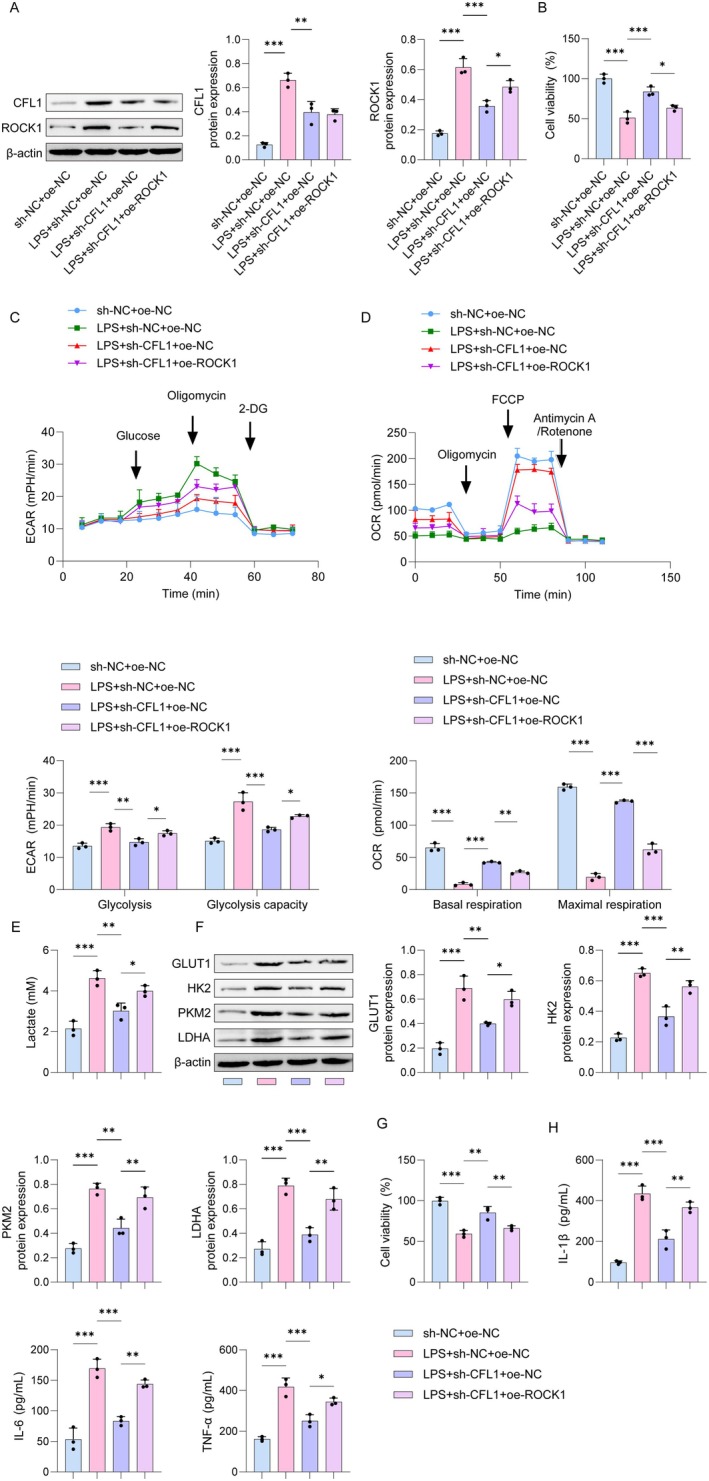
Functional effects of CFL1 and ROCK1 modulation in BV2 cells. (A) Western blot of CFL1 and ROCK1 expression in BV2 cells across groups. (B) Viability of BV2 cells by CCK‐8. (C) ECAR in BV2 cells. (D) OCR in BV2 cells. (E) Lactate levels were measured using a kit. (F) Western blot of GLUT1, HK2, PKM2, and LDHA in BV2 cells. (G) Viability of HT22 cells co‐cultured with BV2 cells. (H) TNF‐α, IL‐1β, and IL‐6 levels in HT22 cells co‐cultured with BV2 cells. Data are presented as mean ± SD. *p < 0.05, **p < 0.01, ***p < 0.001.

BV2 cells were transfected with shCFL1 and/or oe‐ROCK1 and exposed to LPS. Knockdown of CFL1 counteracted LPS‐induced upregulation of CFL1 and ROCK1 (Figure 5A). Furthermore, shCFL1 enhanced cell viability (Figure 5B), decreased ECAR (Figure 5C) and lactate levels (Figure 5E), increased OCR (Figure 5D), and suppressed glycolytic protein expression (Figure 5F) in LPS‐stimulated BV2 cells. These effects were reversed by oe‐ROCK1. Additionally, compared to HT22 cells co‐cultured with the LPS + oe‐NC group, those co‐cultured with BV‐2 cells from the LPS + shCFL1 group exhibited increased viability and decreased levels of inflammatory cytokines. The introduction of oe‐ROCK1 reversed the improvements in viability and inflammatory cytokine levels observed in HT22 cells co‐cultured with BV‐2 cells from the LPS + shCFL1 + oe‐NC group (Figure 5G,H). These results demonstrate that lncA2M‐AS1 regulates microglial glucose metabolic reprogramming and neuroinflammation through the CFL1/ROCK1 pathway.

### 3.6 Overexpression of lncA2M‐AS1 in Vivo Ameliorates PD Phenotypes Through the CFL1/ROCK1 Axis

**FIGURE 6:**
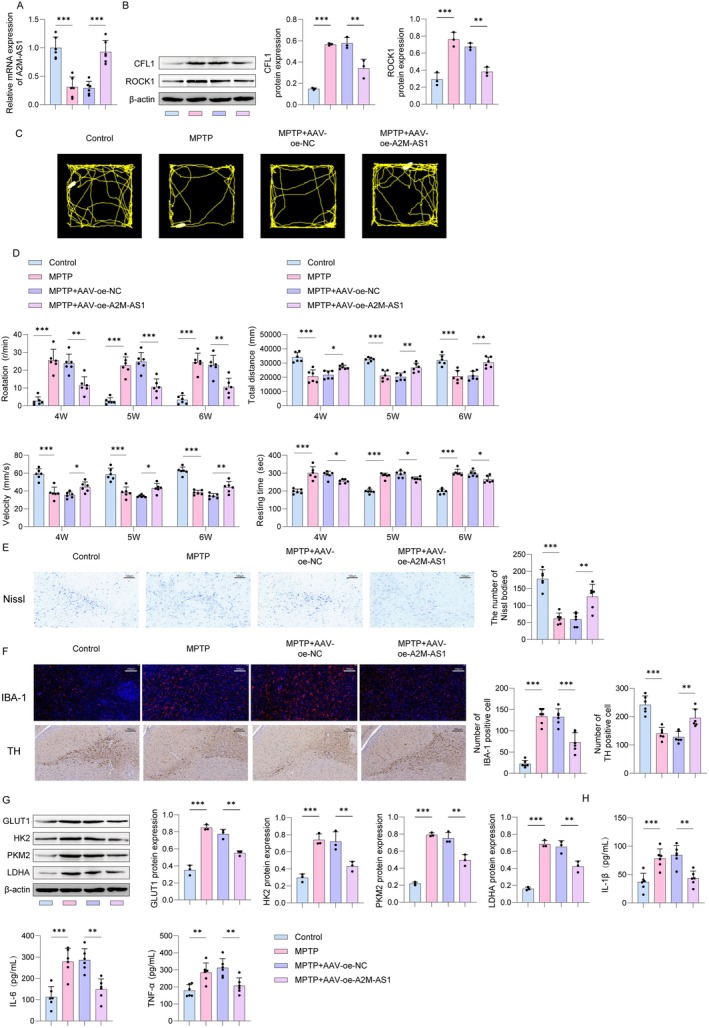
In vivo validation of CFL1 and ROCK1 function in a PD mouse model. (A) qPCR detection of lncA2M‐AS1 in mouse brain tissue. (B) Western blot of CFL1 and ROCK1 in brain tissue. (C) Open field test: Total distance moved, velocity, and resting time within 10 min at weeks 4–6. (D) Apomorphine‐induced rotations at week 4. (E) Nissl staining assessing neuronal damage. (F) IHC of TH and IBA1 in the substantia nigra. (G) Western blot of GLUT1, HK2, PKM2, and LDHA in brain tissue. (H) ELISA measurement of TNF‐α, IL‐1β, and IL‐6. Data are presented as mean ± SD. *p < 0.05, **p < 0.01, ***p < 0.001.

AAV‐mediated overexpression of lncA2M‐AS1 (AAV‐oe‐A2M‐AS1) in PD mice increased its expression in brain tissue (Figure 6A) and reduced CFL1 and ROCK1 levels (Figure 6B). Compared to PD model mice, those treated with AAV‐oe‐A2M‐AS1 exhibited increased movement speed, longer travel distance, shorter resting time, and a significant reduction in apomorphine‐induced rotations (Figure 6C,D). Nissl staining revealed structurally intact neurons with clear nuclear boundaries and abundant blue‐stained Nissl bodies in the control group. In contrast, the MPTP and MPTP + AAV‐oe‐NC groups showed neuronal loss, morphological abnormalities, and a decrease in Nissl‐positive cells. These pathological changes were markedly ameliorated in the MPTP + AAV‐oe‐A2M‐AS1 group, which exhibited improved neuronal morphology, increased neuron number, and more Nissl‐positive cells (Figure 6E). Furthermore, AAV‐oe‐A2M‐AS1 concurrently enhanced TH expression and reduced IBA1 in the substantia nigra of PD mice (Figure 6F), suppressed cerebral glycolysis‐related protein abundance (Figure 6G), and lowered inflammatory cytokine concentrations.

## 4 Discussion

Our previous study, integrating molecular findings with results from clinical samples and animal experiments, reported the critical role of OM‐MSC‐derived exosomal lncA2M‐AS1 in PD neuronal cells [11]. In this study, we further explored how OM‐MSC exosomal lncA2M‐AS1 indirectly influences neuronal cells by reprogramming microglial metabolism, promoting neuroinflammation, and exacerbating PD. These results not only confirm the multifaceted neuroprotective functions of lncA2M‐AS1 but also reveal a novel mechanism by which stem cell exosomes regulate immunometabolism in the PD brain, offering a promising therapeutic target for mitigating neuroinflammation and slowing disease progression.

We first evaluated the regulatory effects of OM‐MSC‐derived exosomal lncA2M‐AS1 on behavioral performance, microglial metabolic reprogramming, and neuroinflammation in PD models. OM‐MSCs have been widely reported to exert neuroprotective functions in various neurological disorders. Previous studies suggested that OM‐MSCs can modulate microglial phenotypes; for instance, Hong et al. demonstrated that OM‐MSCs facilitate M2 over M1 polarization in BV2 microglia, thereby alleviating neuroinflammation and ameliorating Alzheimer's disease pathology [22]. Similarly, Liu et al. reported that hypoxia‐preconditioned OM‐MSCs attenuate microglial pyroptosis in an intracerebral hemorrhage model, resulting in reduced neural damage [23]. However, the mechanisms through which OM‐MSCs or their exosomes regulate microglial function from the perspective of metabolic reprogramming have not been systematically investigated. Notably, a study by Qian et al. revealed that MSC‐derived extracellular vesicles ameliorate autism by targeting microglial glucose metabolism and neuroinflammation [24], which shares certain similarities with the findings of the present study. Similarly, in PD models, we found that lncA2M‐AS1 carried by OM‐MSC exosomes significantly regulates glucose metabolic pathways in microglia and suppresses neuroinflammatory responses. Previous studies have indicated that LPS‐activated microglia shift their metabolic preference from oxidative phosphorylation to glycolysis, polarizing toward a pro‐inflammatory M1 phenotype, which ultimately contributes to neuroinflammation and neurodegeneration [6]. Conversely, inhibition of glycolysis with specific inhibitors markedly reduces the toxicity of activated microglia toward dopaminergic neurons and ameliorates neuroinflammation as well as dopaminergic neuron loss [17], further supporting the therapeutic potential of targeting microglial metabolic reprogramming in PD. Importantly, through specific knockdown of lncA2M‐AS1 in OM‐MSCs, this study confirmed its central role in regulating microglial metabolic reprogramming, suppressing neuroinflammation, and improving behavioral deficits in PD. These results are also consistent with previous reports identifying lncA2M‐AS1 as a potential biomarker in Alzheimer's disease—another neurodegenerative disorder [25], and functional enrichment analyses indicating its involvement in neuronal development and inflammatory signaling transduction [26], further underscoring the critical function of lncA2M‐AS1 in the regulation of neuroinflammation and metabolism.

We next evaluated the relationship between lncA2M‐AS1 and ROCK1. Clinical sample analysis revealed a negative correlation between lncA2M‐AS1 and ROCK1 expression, with ROCK1 being highly expressed in the serum of PD patients. ROCK1 has been previously implicated in PD pathogenesis by inducing apoptosis in dopaminergic neurons [14] and promoting the polarization of microglia toward a pro‐inflammatory phenotype [27], supporting the therapeutic potential of ROCK1 inhibition in neurodegeneration [28]. Furthermore, ROCK1 has been shown to regulate glycolytic processes in valvular interstitial cells [29], endothelial cells [30], and cancer cells [31]; however, its role in microglial metabolic reprogramming remains unexplored. Crucially, we discovered that ROCK1 overexpression reverses lncA2M‐AS1‐induced inhibition of microglial glycolysis and neuroinflammation, pointing to ROCK1 suppression as a mechanism through which lncA2M‐AS1 regulates microglial immunometabolism.

Building upon these findings, we further elucidated the mechanistic link involving the lncA2M‐AS1–CFL1–ROCK1 axis in PD. A well‐documented function of lncRNAs is their ability to orchestrate gene expression at both the transcriptional and post‐transcriptional tiers [32]. We found that lncA2M‐AS1 can directly bind to CFL1 and downregulate its expression. Moreover, overexpression of lncA2M‐AS1 reduced CFL1 protein levels in the brains of PD mice. A plausible explanation is that the binding of lncRNA to mRNA may directly inhibit the translation of CFL1 mRNA [33]. As an actin depolymerizing factor, previous studies on CFL1 have primarily focused on its role in regulating the cytoskeleton and mediating neurotoxicity in neurodegenerative diseases [12]. However, recent evidence has uncovered a novel function of CFL1 in modulating the post‐translational expression of PLD1 by inhibiting ubiquitin‐mediated proteolysis [15]. Consistent with this, our results demonstrated that CFL1 overexpression suppressed ROCK1 ubiquitination and degradation, suggesting that CFL1 may maintain ROCK1 protein stability through a similar post‐translational mechanism. Functional experiments further revealed that ROCK1 overexpression reversed the suppression of microglial glycolysis and the alleviation of neuroinflammation induced by CFL1 knockdown, indicating that CFL1 likely contributes to the regulation of microglial metabolism and inflammation by stabilizing ROCK1 protein.

It should be noted that although the lncA2M‐AS1–CFL1–ROCK1 axis was shown to regulate PD progression in animal and cellular models, its clinical translational value for human PD remains to be confirmed through larger‐scale studies. In addition, future studies using differentiated SH‐SY5Y cells or primary midbrain dopaminergic neurons are warranted to further validate the present findings, as the HT22 cell line used in our in vitro co‐culture experiments does not fully represent human nigral dopaminergic neurons. Second, the specific structural domains through which lncA2M‐AS1 binds to CFL1 mRNA and the precise mechanism by which it regulates translation remain incompletely elucidated; further investigations using techniques such as RNA pulldown and mutant constructs are warranted. Additionally, the upstream and downstream signaling networks of ROCK1 and the mechanisms by which it precisely regulates metabolic enzyme activity in microglia need to be further deciphered. Future studies integrating single‐cell sequencing and metabolomics technologies could systematically reveal the spatiotemporal dynamics of this axis in neuroinflammation and metabolic remodeling. Lastly, peripheral blood serum samples from PD patients were used to detect the expression of lncA2M‐AS1 and ROCK1. However, serum cannot fully reflect the actual status of neurons or glial cells within the central nervous system. Although we validated the key conclusions in in vitro cell and animal models, future studies using cerebrospinal fluid or human brain tissue samples are still needed for further validation.

In this study, exosomes were administered via stereotaxic intracerebroventricular (ICV) injection rather than intravenous injection. The ICV route bypasses the blood–brain barrier, ensures direct CNS delivery with lower doses, and reduces peripheral off‐target effects, making it suitable for mechanism‐of‐action studies [34]. However, it is invasive and less clinically translatable. An alternative is the intranasal route, which is non‐invasive and enables nose‐to‐brain transport. OM‐MSCs are particularly suitable for intranasal delivery given their olfactory mucosal origin. Future studies comparing ICV, intravenous, and intranasal routes will help optimize clinical translation.

In summary, our findings reveal the underlying mechanism through which OM‐MSC‐derived exosomal lncA2M‐AS1 regulates microglial metabolic reprogramming and neuroinflammation through targeting the CFL1–ROCK1 axis. Our results indicate that lncA2M‐AS1 binds to CFL1 mRNA and inhibits its expression, thereby promoting ubiquitination‐mediated degradation of ROCK1 protein, ultimately suppressing microglial glycolysis, alleviating neuroinflammatory responses, and improving behavioral deficits in PD model animals. These findings not only enhance the understanding of how stem cell exosomal lncRNAs regulate the neuro‐immunometabolic microenvironment but also establish a theoretical and experimental foundation for novel PD interventions targeting lncA2M‐AS1 or the CFL1/ROCK1 signaling pathway, paving the way for their future translation.

## Author Contributions

Jiangshan Zhang and Ying Xia guaranteed the integrity of the entire study. Jiangshan Zhang, Guoshuai Yang, and Yujie Hu designed the study and literature research. Yanhui Zhou, Dan Hou, and Ying Xia defined the intellectual content. Jiangshan Zhang, Guoshuai Yang, and Chuang Wang performed the experiment. Yanhui Zhou, Dan Hou, and Ying Xia collected the data. Chuang Wang and Yujie Hu analyzed the data. Jiangshan Zhang and Guoshuai Yang wrote the main manuscript and prepared figures. All authors reviewed the manuscript.

## Funding

This work was supported by the Joint Program on Health Science & Technology Innovation of Hainan Province (WSJK2026MS218).

## Disclosure

The authors have nothing to report.

## Ethics Statement

All experimental protocols were performed in compliance with the National Institutes of Health Guide for the Care and Use of Laboratory Animals and received approval from the Animal Ethics Committee of Haikou People's Hospital (ethics approval no. 2025–309). The study protocol received approval from the Institutional Ethics Committee of Haikou People's Hospital (ethics approval no. 2025–309), and all participants provided written informed consent.

## Consent

The authors have nothing to report.

## Conflicts of Interest

The authors declare no conflicts of interest.

## Data Availability

The data that support the findings of this study are available from the corresponding author upon reasonable request.

## References

[1] K. Kulcsarova , M. Skorvanek , R. B. Postuma , and D. Berg , “Defining Parkinson's Disease: Past and Future,” Journal of Parkinson's Disease 14, no. s2 (2024): S257–s271. 10.3233/JPD-230411 PMC11492139 38489197

[2] D. Weintraub , D. Aarsland , K. R. Chaudhuri , et al., “The Neuropsychiatry of Parkinson's Disease: Advances and Challenges,” Lancet Neurology 21, no. 1 (2022): 89–102. 34942142 10.1016/S1474-4422(21)00330-6 PMC8800169

[3] N. Vijiaratnam , T. Simuni , O. Bandmann , H. R. Morris , and T. Foltynie , “Progress Towards Therapies for Disease Modification in Parkinson's Disease,” Lancet Neurology 20, no. 7 (2021): 559–572. 34146514 10.1016/S1474-4422(21)00061-2

[4] M. Pajares , A. I. Rojo , G. Manda , L. Boscá , and A. Cuadrado , “Inflammation in Parkinson's Disease: Mechanisms and Therapeutic Implications,” Cells 9, no. 7 (2020): 1687. 32674367 10.3390/cells9071687 PMC7408280

[5] S. Isik , B. Yeman Kiyak , R. Akbayir , R. Seyhali , and T. Arpaci , “Microglia Mediated Neuroinflammation in Parkinson's Disease,” Cells 12, no. 7 (2023): 1012. 37048085 10.3390/cells12071012 PMC10093562

[6] H. Yu , Q. Chang , T. Sun , et al., “Metabolic Reprogramming and Polarization of Microglia in Parkinson's Disease: Role of Inflammasome and Iron,” Ageing Research Reviews 90 (2023): 102032. 37572760 10.1016/j.arr.2023.102032

[7] A. Andrzejewska , S. Dabrowska , B. Lukomska , and M. Janowski , “Mesenchymal Stem Cells for Neurological Disorders,” Adv Sci (Weinh) 8, no. 7 (2021): 2002944. 33854883 10.1002/advs.202002944 PMC8024997

[8] S. L. Lindsay , G. A. McCanney , A. G. Willison , and S. C. Barnett , “Multi‐Target Approaches to CNS Repair: Olfactory Mucosa‐Derived Cells and Heparan Sulfates,” Nature Reviews. Neurology 16, no. 4 (2020): 229–240. 32099190 10.1038/s41582-020-0311-0

[9] H. Wang and A. Dwamena , “Olfactory Ecto‐Mesenchymal Stem Cells in Modeling and Treating Alzheimer's Disease,” International Journal of Molecular Sciences 25, no. 15 (2024): 8492. 39126059 10.3390/ijms25158492 PMC11313019

[10] X. L. Zhong , Y. Huang , Y. du , et al., “Unlocking the Therapeutic Potential of Exosomes Derived From Nasal Olfactory Mucosal Mesenchymal Stem Cells: Restoring Synaptic Plasticity, Neurogenesis, and Neuroinflammation in Schizophrenia,” Schizophrenia Bulletin 50, no. 3 (2024): 600–614. 38086528 10.1093/schbul/sbad172 PMC11059802

[11] J. Zhang , C. Wang , G. Yang , Y. Zhou , D. Hou , and Y. Xia , “Olfactory Mucosal Mesenchymal Stem Cell‐Derived Exosome Lnc A2M‐AS1 Ameliorates Oxidative Stress by Regulating TP53INP1‐Mediated Mitochondrial Autophagy Through Interacting With IGF2BP1 in Parkinson's Diseases,” Cell Biology and Toxicology 41, no. 1 (2025): 60. 40111649 10.1007/s10565-025-10009-7 PMC11926059

[12] P. Schönhofen , L. M. Meiros , C. P. Chatain , I. J. Bristot , and F. Klamt , “Cofilin/Actin Rod Formation by Dysregulation of Cofilin‐1 Activity as a Central Initial Step in Neurodegeneration,” Mini‐Reviews in Medicinal Chemistry 14, no. 5 (2014): 393–400. 24813767 10.2174/1389557514666140506161458

[13] T. Landry , D. Shookster , and H. Huang , “Tissue‐Specific Approaches Reveal Diverse Metabolic Functions of Rho‐Kinase 1,” Front Endocrinol (Lausanne) 11 (2020): 622581. 33633690 10.3389/fendo.2020.622581 PMC7901932

[14] Q. Zhang , C. Hu , J. Huang , et al., “ROCK1 Induces Dopaminergic Nerve Cell Apoptosis via the Activation of Drp1‐Mediated Aberrant Mitochondrial Fission in Parkinson's Disease,” Experimental & Molecular Medicine 51, no. 10 (2019): 1–13. 10.1038/s12276-019-0318-z PMC6802738 31578315

[15] B. Yao , Y. Li , T. Chen , et al., “Hypoxia‐Induced Cofilin 1 Promotes Hepatocellular Carcinoma Progression by Regulating the PLD1/AKT Pathway,” Clinical and Translational Medicine 11, no. 3 (2021): e366. 33784016 10.1002/ctm2.366 PMC7982636

[16] R. B. Postuma , D. Berg , M. Stern , et al., “MDS Clinical Diagnostic Criteria for Parkinson's Disease,” Movement Disorders 30, no. 12 (2015): 1591–1601. 26474316 10.1002/mds.26424

[17] J. Cheng , R. Zhang , Z. Xu , et al., “Early Glycolytic Reprogramming Controls Microglial Inflammatory Activation,” Journal of Neuroinflammation 18, no. 1 (2021): 129. 34107997 10.1186/s12974-021-02187-y PMC8191212

[18] J. He , J. Peng , Y. Li , et al., “SENP1 Facilitates OM‐MSC Differentiation Through Activating OPTN‐Mediated Mitophagy to Mitigate the Neurologic Impairment Following ICH,” iScience 27, no. 6 (2024): 109865. 38770132 10.1016/j.isci.2024.109865 PMC11103578

[19] Y. Zhuo , W. S. Li , W. Lu , et al., “TGF‐β1 Mediates Hypoxia‐Preconditioned Olfactory Mucosa Mesenchymal Stem Cells Improved Neural Functional Recovery in Parkinson's Disease Models and Patients,” Military Medical Research 11, no. 1 (2024): 48. 39034405 10.1186/s40779-024-00550-7 PMC11265117

[20] C. Zhang , M. Zhao , B. Wang , et al., “The Nrf2‐NLRP3‐Caspase‐1 Axis Mediates the Neuroprotective Effects of Celastrol in Parkinson's Disease,” Redox Biology 47 (2021): 102134. 34600334 10.1016/j.redox.2021.102134 PMC8487081

[21] C. Lang , C. Yin , K. Lin , et al., “M(6) A Modification of lncRNA PCAT6 Promotes Bone Metastasis in Prostate Cancer Through IGF2BP2‐Mediated IGF1R mRNA Stabilization,” Clinical and Translational Medicine 11, no. 6 (2021): e426. 34185427 10.1002/ctm2.426 PMC8181202

[22] C. G. Hong , M. L. Chen , R. Duan , et al., “Transplantation of Nasal Olfactory Mucosa Mesenchymal Stem Cells Benefits Alzheimer's Disease,” Molecular Neurobiology 59, no. 12 (2022): 7323–7336. 36173534 10.1007/s12035-022-03044-6

[23] J. Liu , J. He , Y. Huang , et al., “Hypoxia‐Preconditioned Mesenchymal Stem Cells Attenuate Microglial Pyroptosis After Intracerebral Hemorrhage,” Ann Transl Med 9, no. 17 (2021): 1362. 34733914 10.21037/atm-21-2590 PMC8506532

[24] Q. Qin , L. Fan , X. Zeng , et al., “Mesenchymal Stem Cell‐Derived Extracellular Vesicles Alleviate Autism by Regulating Microglial Glucose Metabolism Reprogramming and Neuroinflammation Through PD‐1/PD‐L1 Interaction,” J Nanobiotechnol 23, no. 1 (2025): 201. 10.1186/s12951-025-03250-z PMC11895333 40069859

[25] F. Li , Z. Lin , and G. Tian , “Comprehensive Analysis of lncRNA‐miRNA‐mRNA Regulatory Networks for Alzheimer's Disease,” Acta Neurobiologiae Experimentalis (Wars) 82, no. 3 (2022): 263–272. 10.55782/ane-2022-025 36214709

[26] L. Zillich , E. Poisel , F. Streit , et al., “Epigenetic Signatures of Smoking in Five Brain Regions,” J Pers Med 12, no. 4 (2022): 566. 35455681 10.3390/jpm12040566 PMC9029407

[27] C. Barcia , C. M. Ros , V. Annese , et al., “ROCK/Cdc42‐Mediated Microglial Motility and Gliapse Formation Lead to Phagocytosis of Degenerating Dopaminergic Neurons in Vivo,” Scientific Reports 2 (2012): 809. 23139861 10.1038/srep00809 PMC3492875

[28] C. M. Chong , N. Ai , and S. M. Lee , “ROCK in CNS: Different Roles of Isoforms and Therapeutic Target for Neurodegenerative Disorders,” Current Drug Targets 18, no. 4 (2017): 455–462. 27033194 10.2174/1389450117666160401123825

[29] H. Liu , H. Yin , Z. Wang , et al., “Rho A/ROCK1 Signaling‐Mediated Metabolic Reprogramming of Valvular Interstitial Cells Toward Warburg Effect Accelerates Aortic Valve Calcification via AMPK/RUNX2 Axis,” Cell Death & Disease 14, no. 2 (2023): 108. 36774349 10.1038/s41419-023-05642-1 PMC9922265

[30] K. Yang , X. Q. Zhou , K. Guo , et al., “Diosgenin in Dioscorea Spongiosa Suppresses Glycolysis‐Driven Angiogenesis as a ROCK1 Inhibitor,” Journal of Agricultural and Food Chemistry 73, no. 17 (2025): 10214–10229. 40252032 10.1021/acs.jafc.4c11678

[31] A. G. Alkhathami , A. S. Sahib , M. S. al Fayi , et al., “Glycolysis in Human Cancers: Emphasis circRNA/Glycolysis Axis and Nanoparticles in Glycolysis Regulation in Cancer Therapy,” Environmental Research 234 (2023): 116007. 37119844 10.1016/j.envres.2023.116007

[32] A. B. Herman , D. Tsitsipatis , and M. Gorospe , “Integrated lncRNA Function Upon Genomic and Epigenomic Regulation,” Molecular Cell 82, no. 12 (2022): 2252–2266. 35714586 10.1016/j.molcel.2022.05.027 PMC9219586

[33] R. Cai , Y. Sun , N. Qimuge , et al., “Adiponectin AS lncRNA Inhibits Adipogenesis by Transferring From Nucleus to Cytoplasm and Attenuating Adiponectin mRNA Translation,” Biochimica et Biophysica Acta ‐ Molecular and Cell Biology of Lipids 1863, no. 4 (2018): 420–432. 29414510 10.1016/j.bbalip.2018.01.005

[34] A. J. Atkinson, Jr. , “Intracerebroventricular drug administration,” Transl Clin Pharmacol 25, no. 3 (2017): 117–124. 32095461 10.12793/tcp.2017.25.3.117 PMC7033376

